# Supplementary material for: Perinatal and early life risk factors of adverse early childhood developmental outcomes: Protocol for systematic review using socioecological model
Source: PLoS One. 2024 Oct 17;19(10):e0311500. doi: 10.1371/journal.pone.0311500 (PMC11486404; doi:10.1371/journal.pone.0311500)
Supplement: S2 Table — (DOCX) [file pone.0311500.s002.docx]

**S2 Table: Search strategy**

**Title: Perinatal and early life risk factors of adverse child developmental outcomes: Systematic review**

**Databases – Medline (PubMed), Embase, Global Health, PsycINFO, CINAHL, Web of Science**

|  | **Population:** Children | **Outcomes:** child developmental outcomes | ***Exposures: P****erinatal and early life risk factors* |
| --- | --- | --- | --- |
| **Keywords** | child* OR toddler* OR preschool* OR "pre-school*" OR kindergarten* OR infant* OR baby OR babies OR “school age” OR “year five” OR “year four” OR year three” OR "early childhood" OR “school readiness” | development* OR neurodevelopment* OR “developmental outcome*” OR “developmental adversit*” OR “developmental vulnerability” OR “developmental difficult*” OR “developmental problem*” OR “development* disorder*” OR “developmental regression*” OR “developmental delay*” | *(Risk factor*" OR "predictor*"*  *OR*  *Sociodemographic* OR "maternal age" OR sex OR educational status* OR income OR* “*sociodemographic disadvantage*” OR remoteness OR Indigenous* *OR*  *Alcohol OR substance abuse OR Smoking OR “screen time” OR Drug OR preterm OR “gestational age” OR premature* OR “low birth weight” OR LBW or parity or gravidity* OR “*pregnancy complication” OR preeclampsia OR eclampsia OR “APGAR score” OR “Inter-pregnancy interval” OR “Interpregnancy interval” OR mental health OR depression OR anxiety OR corticosteroid OR surgery OR hospitalisation OR malnutrition OR infectious diseases OR chronic diseases OR hypertension OR medication OR* *anaemia OR BMI OR “body mass index” OR Temperature OR hot OR heat OR cold OR ‘climate change’ OR heatwave OR coldwave OR ‘air pollution’ OR “particulate matter” OR ‘fine particle’ OR ‘gaseous pollutant’ OR ‘air pollutant’ OR “PM2.5” OR PM10 OR ‘nitrogen dioxide’ OR ‘sulfur dioxide’ OR ‘sulphur dioxide’ OR ‘nitrogen oxide’ OR ‘carbon monoxide’ OR ozone OR SO2 OR NO2 OR O3 OR CO)* |
