# Supplementary material for: Perinatal and early life risk factors of adverse early childhood developmental outcomes: Protocol for systematic review using socioecological model
Source: PLoS One. 2024 Oct 17;19(10):e0311500. doi: 10.1371/journal.pone.0311500 (PMC11486404; doi:10.1371/journal.pone.0311500)
Supplement: Attachment — Submitted filename: Point by point response.docx [file pone.0311500.s004.docx]

**Ref: PONE-D-23-35644**

Dr Jennifer Tucker,

**Editor,** **PLOS ONE**

**Title:** Perinatal and early life risk factors of early childhood developmental vulnerability: Protocol for systematic review and meta-analysis.

**Authors:** Kendalem Asmare Atalell, Gavin Pereira, Bereket Duko, Sylvester Dodzi Nyadanu, Gizachew A Tessema

**Subject: Submission of revised manuscript**

Dear Editor,

Thank you for the opportunity to revise our manuscript “Perinatal and Early Life Risk Factors of Early Childhood Developmental Vulnerability: Protocol for Systematic Review and Meta-analysis”. We thank the reviewers and editors for their constructive and insightful comments and suggestions. We have now revised the manuscript and incorporated the comments. Our responses are given in point-by-point responses to each reviewer and the editor’s comments below. We have also attached the revised manuscript with tracked changes and a clean version.

We hope the revised version is suitable for publication and look forward to hearing from you soon.

Sincerely

Kendalem Asmare Atalell

Corresponding Author

**Response to the editor and reviewers’ comments,**

**Response to the editor**
**Comment #1:** Specifically, the reviewer requests clarification around the methodology, such as the provision of an explanation (and citation) for the socioecological model and additional background information, and suggests reconsidering the suggested subgrouping, which may be too broad.

**Response:** As requested by reviewers, we have now revised the manuscript to clarify the methodology and provide additional explanation for the socio-ecological model on *page 8 lines 219-232* on the revised manuscript. *“The socio-ecological model is important to map the complex interplay between various biological, psychological, behavioural, perinatal and environmental risk factors of adverse early childhood developmental outcomes into multilevel factors that include intrapersonal, interpersonal, community and societal levels, 50 that help to target health promotion activities at the individual, family, community or societal level. The four-level socioecological model will be formed based on the preliminary literature review. The first level is the intrapersonal risk factors or individual/child-related risk factors, which include characteristics that arise from the child, such as child age, sex, nutrition status, comorbid conditions, etc. The second level of risk factors is interpersonal/family or caregiver, which is related to maternal and paternal risk exposures. The third level of risk factors is community-related risk factors related to neighbourhoods, schools, peers, early childhood education and care centres. The final fourth level of risk factors are societal risk factors, including policy, program, system, culture and climatic risk factors.”*

**Journal Requirements**

**Response:** We have revised the manuscript to meet the PLOS ONE Journal requirements. We have also added a data availability section in the revised version of the manuscript *(Page 10, Lines 287 & 288)*.

“Data availability.

All relevant data are included in the manuscript.”

**Response to the reviewer's comment**

**Review #1 Comments to the Author**

**Comment 1**. This is a fair protocol but some details are missing. Adding that clarity now will make the review process easier. There are many studies on this topic so a really comprehensive systematic review, done well, is a helpful contribution to the field. However there are similar entries on PROSPERO, see: here: https://www.crd.york.ac.uk/prospero/display_record.php?RecordID=3215

**Response:** Thank you for your concern and for providing a similar review protocol on the topic. We aim to provide a comprehensive systematic review mapping the available evidence to date. Unfortunately, we couldn’t find the above PROSPERO registered protocol. But, when we explore it now, it was published in 2012 (i.e., 12 years ago), and still, we couldn’t find the actual systematic review. However, no recent systematic review has provided a comprehensive and in-depth assessment of the literature. Our review will also include adverse developmental outcomes namely developmental delays and developmental vulnerability outcomes, which have not been included in the existing systematic review. We also plan to explore all available risk factors for child development outcomes.

Comment #2. Please ensure your contribution is a novel contribution to the field. Your registered PROTOCOL uses the word 'adversities' rather than 'vulnerabilities'. The terms need to be consistent.

**Response:** We have now revised the wording to avoid confusion. Hence, considering the outcomes to be included, we have now revised the title to “Perinatal and early life risk factors of adverse early childhood developmental outcomes: Protocol for systematic review and meta-analysis”.

Comment #3. Introduction
The concept of early childhood development vulnerability needs to be explained early on. Please define this and if it has been derived from other sources please cite accordingly.
Other studies that have looked at the impact of development at school entry and trajectories of development on adolescent health and may be worth a look if you are making a life course argument <https://www.mdpi.com/1660-4601/18/21/11613>
and <https://www.jpeds.com/article/S0022-3476(23)00474-2/fulltext> and want to cite the most recent research.
Classification system for child development is also discussed here: <https://www.mdpi.com/1660-4601/18/21/11613>

**Author response:** We then revised the suggestions and shared important materials. We have revised the introduction and cited relevant literature to support our definition of the outcomes which included developmental vulnerability and developmental delays until the age of 8 on *page 3 lines 71-87* and presented as follows:

“*Adverse Early child developmental outcomes refer to difficulties or delays in various areas of child development during early childhood periods, including challenges or disruptions in various domains of development, such as physical, cognitive, language, motor, social-emotional, and behavioural areas. Several assessment tools are used to assess early childhood development, such as the Early Development Instrument (EDI), a widely used tool for assessing developmental status at school entry. It assesses child development in five domains: physical health and well-being, social competence, emotional maturity, language and cognitive development, communication skills, and general knowledge. Child development can also be assessed using Ages and Stages Questionnaires (ASQ), which cover communication, gross motor, fine motor, problem-solving and personal social skills.* *Bayley Scales of Infant and Toddler Development (Bayley), Denver Developmental Screening Test (DDST), Peabody Developmental Motor Scales (PDMS), and Social-emotional assessment tools are also among some of the early childhood developmental assessment tools. These tools vary in format, administration, target age groups and developmental area classifications. Understanding the various domains of early childhood developmental outcomes and their risk factors can inform policy and program development at the community and government levels to better support early childhood development.”*

Comment #4. Please provide a simple explanation (and citation) for the socioecological model.

**Response:** We have now provided citations and a simple explanation of how we apply the socioecological model in our systematic review on *page 4, lines 97-106, and page 8, lines 219-232*.

*“The various perinatal and early childhood risk factors will be summarized using a four-level socioecological model to better understand early childhood development adversities and potential prevention strategies. The socioecological model is a theoretical framework used to understand and analyze the complex interaction between intrapersonal (individual child characteristics), interpersonal (family/caregiver related characteristics), community (school, peers, teachers, daycares, and neighborhoods related characteristics), and societal (climatic, cultural, systems, policy, and program-related characteristics) level factors28 (Fig 1). The socio-ecological model is an important approach to summarize the various risk factors, which leads to recommend a targeted intervention at each level.”*
comment # 5: The existing evidence on what is known in this field is more comprehensive that the lines portrayed: There were some fragmented systematic syntheses of evidence such as child developmental outcomes associated with low birth weight and small for gestational age in Indigenous Children from Australia, Canada and New Zealand.
Please provide a brief but comprehensive summary on what is known about risk factors (state which ones) that affect ECD vulnerability and state which countries. (This is why your definition of ECD vulnerability is needed right from the outset).

**Response:** Thank you for your insightful suggestion. We have now summarised the existing systematic review on *page 4 lines 112 -126* and defined our outcomes (early childhood outcomes and developmental delays) on *page 3 lines 71-87( see the response for comment #3)*:

*“Previous systematic synthesis have been conducted to assess the risk factors of early childhood developmental outcomes. 27 35-38 However, those studies used either single exposure or a single domain of early childhood developmental adversities. Thus, a comprehensive synthesis of the multilevel risk factors towards adverse early childhood developmental outcomes is limited.39 On the Other hand, the current study will summarize risk factors of adverse early childhood developmental outcomes into individual, interpersonal, and community-level risk factors. In addition, we include all the developmental domains, including physical, social, cognitive, language, communication and other domains based on each assessment tool report. Hence, estimating the global burden of adverse early childhood developmental outcomes and identifying the multilevel associated risk factors is imperative for identifying vulnerable populations and implementing targeted health promotion activities at each level.8 Therefore, this systematic review aimed to estimate the global burden of early childhood developmental vulnerabilities and comprehensively map the broader risk factors of early childhood developmental vulnerability using the socioecological model.”*
Comment #6. Research Qs - is research question 2 about predictive risk factors?

**Response:** Yes, it is about predictive risk factors of adverse early childhood developmental outcomes.
Comment 7. Eligibility: This section needs to be much clearer. The population blurs with the Concept. Suggest: Population (children 0-5), Exposure (risk factors), Outcomes (developmental) and Study types. See here: <https://systematicreviewsjournal.biomedcentral.com/articles/10.1186/s13643-021-01694-6>

**Response:** Thank you for your suggestion. As suggested, we have revised the eligibility criteria to make them clearer on *pages 5 and 6 lines 138-166*. *“We used the Population, Exposure, and Exposure Comparison, Outcome and Studies (PECOCS) criteria to select eligible studies for our study in the following format.*

*1)* ***Population:*** *Children underwent early childhood developmental assessments under 8 years old. The age of 8 years is selected because early childhood is defined as the period from prenatal development to eight years, according to WHO.*

*2)* ***Exposure:*** *The exposure of this study includes the various perinatal and early life risk factors. Perinatal and early life risk factors include biological, psychosocial, behavioural, and environmental risk factors associated with early childhood developmental outcomes.*

*3)* ***Comparison:*** *Children with no or low levels of exposure to the risk factors will be used as a comparator for this study.*

*4)* ***Outcomes:*** *The primary outcome of this systematic review is adverse early childhood developmental outcomes such as adverse outcomes of physical, cognitive, social, emotional, language and neurological developments assessed through various assessment tools such as Early Development Instrument (EDI), Age and Stages Questionnaire (ASQ), Bayley Scales of Infant and Toddler Development (Bayley), Denver Developmental Screening Test (DDST), Peabody Developmental Motor Scales (PDMS), and Social-emotional assessment tools etc.*

*5)* ***Studies:*** *We will include observational studies such as cohort, case-control, nested case-control, and cross-sectional studies reporting the prevalence or incidence of adverse early childhood developmental outcomes and associated risk factors in children. There will be no restriction based on country of origin or geographical location except language (only published in English) from January 2000. The year 2000 was chosen due to the change in technology, social media use, social and cultural contexts, early childhood education, and environmental exposures over time, which makes it important to incorporate more recent and up-to-date knowledge in the field. There have been substantial changes in policies, interventions, and programs over the last two decades, all aimed at addressing early childhood developmental outcomes.”*
comment #8: The risk factors proposed here need to be introduced more clearly in the introduction.

**Response:** In the revised version, we have described the broad range of risk factors in the introduction section on *pages 3 and 4 lines 90-97*. *“Previous studies showed that early childhood developmental outcomes were affected by various perinatal and early life risk exposures such as genetic and biological factors, prenatal conditions such as maternal health, nutrition, exposure to toxic substances, prenatal care during pregnancy, birth complications, child health, parenting behaviours, access to early childhood education and care, socioeconomic disadvantages, and environmental exposure to toxins or pollutants. A systematic review conducted among Indigenous Children from Australia, Canada and New Zealand showed that child developmental outcomes were associated with low birth weight and small for gestational age”*
Comment # 9: Data Synthesis: The terms domains and measurement tools are suddenly introduced. Please be consistent in language and terms throughout the document.

**Author response:** We have introduced child developmental domains and developmental assessment tools in the introduction section of the revised manuscript *(see page 3, lines 71-87) (this part is presented in the response to comment #3).*

Comment #10: It states you expect a variety of early child developmental outcome domains; - why has the language changed from ECD vulnerabilities to developmental outcomes?

**Author response:** Thank you for your concerns. Given that child developmental outcomes encompass both child developmental vulnerabilities and developmental delays, we have now preferred to use the term child developmental outcomes to facilitate our comprehensive evaluation of both outcomes. Accordingly, we have now revised the title and aim of the study, as well as clearly defined outcomes.

Comment #11: Your search strategy only lists negative developmental outcomes. Again - this must all be clarified in the eligibility section and then all the terms must remain consistent. E.g. - is the outcome vulnerability (sub-optimal development) or any developmental domain?

**Author response:** Our primary aim is the adverse developmental outcome for early detection and prevention activities. As some studies will likely report risk factors for one or more sub-domains, we plan to collate the risk factors for sub-outcomes. As a result, we have ensured that our search strategy included keywords for developmental domains.
Comment #12: It appears there is narrative and quantitative synthesis. Please describe both clearly including a section on confidence in cumulative evidence (i.e. for any narrative synthesis - how will you determine the strength of the evidence?)

**Author response:** We have revised the data synthesis section as follows *(see pages 7 & 8 lines 214-240*): *“A textual and narrative synthesis of the global burden of early childhood developmental outcomes will be conducted in each developmental domain, such as physical, social, emotional, cognitive, language, communication, etc. and assessment tools. Thus, a standard definition will be developed for adverse early childhood developmental outcomes by reviewing the literature in the early stage of the data extraction process. Along with the textual or narrative synthesis, a four-level socioecological model or framework will be used to synthesise the risk factors of early childhood developmental adversities. The socio-ecological model is important to map the complex interplay between various biological, psychological, behavioural, perinatal and environmental risk factors of adverse early childhood developmental outcomes into multilevel factors that include intrapersonal, interpersonal, community and societal levels, that help to target health promotion activities at the individual, family, community or societal level51. The four-level socioecological model will be formed based on the preliminary literature review. The first level is the intrapersonal risk factors or individual/child-related risk factors, which include characteristics that arise from the child, such as child age, sex, nutrition status, comorbid conditions, etc. The second level of risk factors is interpersonal/family or caregiver, which is related to maternal and paternal risk exposures. The third level of risk factors is community-related risk factors, which are related to neighbourhoods, schools, peers, early childhood education and care centers. The final four risk factors are societal risk factors, including policy, program, system, culture and climatic risk factors. A random-effects meta-analysis will be performed to pool the global burden of adverse early childhood developmental outcomes across studies. Studies that report the prevalence, proportion, or incidence of adverse early childhood developmental outcomes as a whole or in each domain will be polled to estimate the global burden. The risk factors of child developmental outcomes will also pool using random-effect meta-analysis. Studies have reported that odds ratio, relative risk, risk ratio, and regression coefficient will be analysed. Random effect metanalysis assumes heterogeneity of true-effect sizes of early childhood developmental adversities.”*

Comment #13: The subgroup analysis is too broad to be well thought out. Perhaps think about what subgroup analysis would be useful?

**Author response:** We have now revised the subgroup analysis as follows *(see page 9 lines 251-256):*

“*Subgroup analysis will be conducted for developmental vulnerability and developmental delay. A sub-analysis will also be conducted based on the assessment tools. We also plan to conduct a subgroup analysis of developmental domains. The stratified analysis will also be performed based on the study setting, publication year, and demographic factors such as sex, country income levels, and WHO global regions. Sensitivity analyses will also assess the impacts of methodological quality, sample size and analysis methods.”*
14. Can Quality assessment templates and data extraction templates be included as supplementary files?

**Author response:** We have now included the data extraction checklist as a supplement file and use the JBI quality appraisal tools for quality assessment (found here: <https://jbi.global/critical-appraisal-tools>).
