# Supplementary material for: Perinatal and early life risk factors of adverse early childhood developmental outcomes: Protocol for systematic review using socioecological model
Source: PLoS One. 2024 Oct 17;19(10):e0311500. doi: 10.1371/journal.pone.0311500 (PMC11486404; doi:10.1371/journal.pone.0311500)
Supplement: Attachment — Submitted filename: Point by point.docx [file pone.0311500.s005.docx]

**Ref:** PONE-D-23-35644R1

Dr Jennifer Tucker,

**Editor, PLOS ONE**

**Title:** Perinatal and early life risk factors of adverse early childhood developmental outcomes: Protocol for systematic review and meta-analysis.

**Authors:** Kendalem Asmare Atalell, Gavin Pereira, Bereket Duko, Sylvester Dodzi Nyadanu, Gizachew A Tessema

**Subject: Submission of revised manuscript**

Dear Editor,

Thank you for the opportunity to revise our manuscript for the second time, “Perinatal and early life risk factors of adverse early childhood developmental outcomes: Protocol for systematic review and meta-analysis.” We are pleased to revise the manuscript and incorporate the comments. Our responses are given in point-by-point responses to each reviewer and the editor’s comments below. We have also attached the revised manuscript with tracked changes and a clean version.

We hope the revised version is suitable for publication now and look forward to hearing from you soon.

Sincerely

Kendalem Asmare Atalell

Corresponding Author

**Response to Reviewers' comments**

**Response:** Thank you for your valuable insights. We aim to provide a comprehensive systematic review identifying various perinatal and childhood risk factors for adverse developmental outcomes.

**Response to Reviewer #2**

1. Thank you for the opportunity to review this interesting paper, which proposes to conduct a broad and comprehensive systematic review of the literature on early childhood developmental vulnerabilities and perinatal and early life risk factors. This review is much needed and will be a useful tool to researchers studying early child development. The methodological strategy overall is robust and well explained. My main concern is regarding the definition and description of early childhood developmental vulnerability and the perinatal and early life risk factors. These are quite broad and potentially overlapping. The authors will find it difficult to extract meaningful information if they don’t better define and delineate these. It might be useful to attempt to extract a few papers as a pilot exercise to see whether it is feasible. The review is ambitious, and unless they narrow down what they are including in terms of their outcome and exposure, I am concerned about feasibility and clarity of the resulting tables.

**Response:** Thank you for your interest in our comprehensive review; we are very grateful for your valuable concerns. The outcome and exposure are now well explained to clarify what we want to do in our review in the revised manuscript, page 5 lines, lines 129-144: *“Exposure: The exposures in this study include various perinatal and early childhood risk factors, such as biological, sociodemographic, child health and medical history, childhood nutrition, maternal mental health issues, pregnancy-related maternal health conditions, maternal nutrition, prenatal exposure to substances and pollutants, environmental pollutants, and climatic factors.*

*Outcome: The primary outcome of this systematic review is adverse early childhood developmental outcomes, such as physical, cognitive, language and communication and social-emotional developments,43 44 which is defined as difficulties or delays in various areas of child developmental vulnerability or developmental problems at composite or sub-domain levels.”*

2. The Concept section (#2 page 4) talks about a wide range of risk factors, and it is not until the data synthesis section that we get a sense of what these mean. Recommend having this come earlier.

**Response:** We have revised the criteria from PCC to PECOS and the risk factors were introduced in the exposure section in the revised version of the manuscript. (See response #1).

3. The authors should better define or perhaps consider narrowing down the early risk exposures. I am especially concerned with including behavioural risk factors and then including behavioural problems in the developmental vulnerability assessments. Many of the measures of the EDI for example includes early life behavioural and psychosocial risk factors. Since the authors are including such a wide range of developmental outcomes (5 or 6? “outcomes of physical, cognitive, social, emotional, language and neurological developments”) it might be better to narrow down the exposures of perinatal risk. If leaving all of these in, they should be clearly defined early on beyond this: “Perinatal and early life risk factors include biological, psychosocial, behavioural, and environmental risk factors associated with early childhood developmental outcomes.” However, I am still concerned that the number of factors to consider in all of these would be absolutely massive, from prenatal smoking, prenatal substance use, low birth weight, parental mental illness/offending, socio-economic disadvantage, neighbourhood crime, child maltreatment, ACEs, early behavioural problems etc. All of these examples could potentially fit in those categories. I am struggling to see how this would be feasible in one systematic review.

**Response:** Thank you again for your valuable comments. We also noted that it is a very wide. However, our aim is to summarize all the factors affecting early childhood developmental outcomes using the socioecological model as a framework to better recommend comprehensive and effective health promotion activities at the individual, family, community and societal levels. Hence, we will map all the risk factors of developmental adversities in early childhood.

**Response to Reviewer #3:**

1. Thank you for the opportunity to review this protocol paper. I note that it has already been revised based on one set of peer review, and I am grateful to have access to those comments and responses in order to see the paper’s journey thus far.

Overall the paper is not very clearly written, and I share concerns of the original reviewers that there is a lack of clarity around the case for this systematic review in terms of its unique contribution, and in terms of the articulation of the theoretical basis. Whilst I can see improvements have been made in response to initial review, I am not sure all points have been adequately addressed and there is still much to do to improve this paper sufficiently for publication. I will make specific points, major and minor, below.

**Response:** Thank you so much for your thoughtful comments

Abstract

2. Line 31 – I’m not sure about ‘multifacetedly’ as a word. Suggest remove it or express in more simple terms.

**Response:** We have now revised the wording to avoid confusion.

3. Line 41-42 – why is ‘general knowledge’ being termed as a developmental outcome?

**Response:** We have revised the sentence to make it clear for readers as follows developmental outcomes domains in the revised version of the manuscript. The sentence is revised as follows “*We will systematically search for articles on adverse early childhood developmental outcomes, which include physical, cognitive, language and communication and social-emotional development* *from main databases, such as EMBASE, Medline, Global Health, PsycINFO, CINAHL and Web of Science Core Collection from January 2000 to January 2024.”* Page 2 lines 37-41.

4. Line 48 – should the outcome not be expressed simply as ‘developmental outcome’ rather than ‘adverse early childhood developmental outcomes’?

**Response:** Thank you, our outcome is developmental outcome in early childhood periods (under eight years of age). It can be either adverse outcomes such as delay or vulnerability or optimal outcomes when the child reaches his/her potential for his or her age. However, our aim in this systematic review is to identify the risk factors associated with adverse developmental outcomes, which is why we prefer to say adverse early childhood developmental outcomes.

5. Lines 35 & 52 – the former refers to ‘the’ socioecological model, the latter to ‘a’ socioecological model. It is not clear from this exactly which model is being applied, and whether a specific approach is being taken.

**Response:** We have revised it and consistently used the socioecological model. The socio-ecological model is briefly introduced in the introduction as well as in the data synthesis section of the manuscript on page 4 lines 101-108 “The socio-ecological model is a theoretical framework used to understand and analyze the complex interaction between 1) Individual factors: factors arise from the individual child such as genetics, health status, and personal behaviours. 2) Interpersonal factors: maternal, paternal, or household-related influences, including family dynamics and parenting styles. 3) Community factors: school, peers, teachers, daycare environments, and neighborhood-related influences. 4) Societal factors: broader influences include climatic conditions, cultural norms, systems, policies, and programs.”

6. Line 56 – suggest ‘in the public domain’ rather than ‘from the evidence base’.

**Response:** Thank you, Corrected

7. Line 57 – suggest ‘developmental outcomes’ would be a better keyword than ‘developmental vulnerability’.

**Response:** We have changed the keyword from developmental vulnerability to developmental outcome; thank you.

Introduction

8. I found the introduction generally lacked a coherent structure and would benefit from revision. The decision to go into detail about a small group of measures from line 74-87 is curious and oddly specific. The sentence beginning ‘these tools vary in format…’ at line 83-84 is the important point, and this should be the main driver of this paragraph.

**Response:** Thank you for the suggestions; we have made a major change in the introduction section, and we believe the introduction is now improved well (See pages 3 and 4.

9. At line 97, the sentence beginning ‘The various perinatal…’ is premature as the model has not yet been described. In relation to the model, I find it odd that Bronfenbrenner has not once been cited, and that the citations used around the model are often not primary. Further, Figure 1 is rudimentary and inadequate to illustrate the strength of this model. There are many examples online where examples of the type of factor at each layer are given, and this would certainly be a good approach for a paper offering such a comprehensive review.

**Response:** We have made changes in the introduction and this section is also removed and updated with more suitable descriptions. Thank you.

10. On line 95 you refer to a review regarding indigenous children – it is not clear why this very specific example was chosen to illustrate your point.

**Response:** We have removed this specific example from the revised manuscript since our review is not solely based on indigenous populations.

11. Line 111 – ‘accessing the quality early childhood’ should be ‘accessing quality early childhood’

**Response:** Corrected

12. Line 113 – plural syntheses should be used instead of synthesis.

**Response:** Corrected

13. Line 123 – suggest paragraph break at ‘therefore’ (although would then revise to not begin sentence with a conjunction).

**Response:** Corrected

Research questions

14. I share the concerns of previous reviewers about the clarity and novelty of these. For example, RQ1 refers to a ‘global estimate’ but it is entirely unclear what metric will be used.

**Response:** We have revised the research question as follows: “What are the perinatal and early childhood risk factors for adverse early childhood developmental outcomes?” page 4, *lines 115-117.*

Method

15. Line 133 – PRISMA is missing the I

**Response:** Corrected

16. Line 144 – under Exposure, why has the term Adverse Childhood Experience (ACE) not been used in searches? Also, you need to stipulate the timing of exposure in relation to outcome.

**Response:** Thank you. We have done a preliminary literature review before developing the search term, and adverse childhood experience is a general term to be included in the search term. Regarding the timing of the exposure, we plan to include all the exposure time, including the perinatal and childhood exposure.

17. Lines 152-155 – again, very specific examples of potential measures. It’s not clear why.

**Response:** There are many developmental assessment tools; we list a few as examples, which are commonly used in many studies as per our preliminary literature review.

18. Line 156 – Studies – you refer to designs used – what will you do to ensure/assess that samples are population representative (as many studies will be clinical populations and so skew any findings)?

**Response:** The data extraction checklist included the study setting (clinical or community), data source, and population, which can identify which study is conducted in a clinical population. We plan to conduct a subgroup analysis based on the available data.

19. Line 161-164 – this is a perfect opportunity to link to the ‘chronosphere’ dimension of Bronfenbrenner’s model.

**Response:** Bronfenbrenner’s model is more focused on the developmental context of children and the interplay of nested environmental systems, whereas the socioecological model is broader, focusing on health behaviors and outcomes incorporating a more extensive range of social and policy influences. Hence, we plan to use the socioecological model or framework rather than Bronfenbrenner’s model.

20. Line 172-184 – make it clear that this is your core search strategy, and that specific iterations tailored to each database are available in supplementary material.

**Response:** We have revised this section to make it clear that this is the core search strategy on page 6 lines 173-175.

21. Line 199 – will you also record the age @ exposure?

**Response:** Initially, we plan to collect age at the exposure; after the preliminary literature review, we excluded it because most of the studies are long cohort studies that start in perinatal to childhood, which makes it difficult to capture age exposure. However, we will record age at developmental assessment.

22. Line 205 – you mention two authors will extract data – will this be double entry from all papers, or a shared workload (i.e., 50:50 split)?

**Response:** We have revised as follows: “Given the anticipated large volume of studies in our systematic review, we plan to conduct double data extraction for approximately 20% of the included studies. After ensuring consistency in the extracted data, we will proceed to extract the remaining data, with each author handling 50% of the remaining studies.” Page 7 lines 195-199.

23. Line 209 – citation 48 is to a secondary source, and the link to the primary is broken. Please update.

**Response:** We have now updated the citations.

24. Line 216-217 – you write ‘a standard definition will be developed for adverse early childhood developmental outcomes’ as part of your synthesis – but surely this will be developed as part of your inclusion criteria?

**Response:** Thank you so much for your insights. This section has also been revised, and the outcomes are already defined in the inclusion criteria. (See response #1).

25. Line 225 – you write ‘the four-level socioecological model will be formed based on preliminary literature review’ – what exactly do you mean by this?

**Response:** In the socioecological model, the multilevel factors are customised based on the nature of the problem and the data. After doing a preliminary literature search. We customise the socioecological model to four levels. 1) Individual level: is more child level risk factors 2) Interpersonal risk factors are related to maternal, paternal and household level risk factors 3) Community-level risk factors include organizational risks such as school, preschool, and daycare, as well as community and environmental-related risk factors. 4) Societal-level risk factors are risk factors related to national programs, systems and policy-related risk factors for child developmental adversities. This is elaborated in the revised manuscript on page 8, lines 218-226.

26. Line 233 – you write that you will pool the global burden. Again, using what metric?

**Response:** Thank you so much. We dropped these research questions based on our preliminary literature search result, we couldn’t be able to get data for the global estimate.

27. Line 236 – polled should read pooled.

**Response:** This section has already been removed.

28. Regarding the plans for analysis, as statistical analysis is not my specialism I am not in a position to comment on these.

Overall this protocol may eventually be publishable, but I find it difficult to assess this with the lack of specificity in articulation of the research questions and methods.

**Response:** Thank you so much for your recommendation; we believe the paper has now become clearer.
