# Supplementary material for: Perinatal and early life risk factors of adverse early childhood developmental outcomes: Protocol for systematic review using socioecological model
Source: PLoS One. 2024 Oct 17;19(10):e0311500. doi: 10.1371/journal.pone.0311500 (PMC11486404; doi:10.1371/journal.pone.0311500)
Supplement: Attachment — Submitted filename: Response to reviewers.docx [file pone.0311500.s006.docx]

**Response to reviewers’ comments**

PONE-D-23-35644R2

Perinatal and early life risk factors of adverse early childhood developmental outcomes: Protocol for systematic review using socioecological model.

Dear Editor,

Thank you again for the opportunity to revise our manuscript, “Perinatal and early childhood risk factors of adverse early childhood developmental outcomes: Protocol for systematic review using socioecological model.” We are pleased again to revise the manuscript, which makes improvements. Our responses are given in point-by-point responses to each reviewer and the editor’s comments below. We have also attached the revised manuscript with tracked changes and a clean version.

We hope the revised version is suitable for publication now and look forward to hearing from you soon.

Sincerely

Kendalem Asmare Atalell

Corresponding Author

**Response to reviewers’ comment**

Journal Requirements

Please review your reference list to ensure that it is complete and correct. If you have cited papers that have been retracted, please include the rationale for doing so in the manuscript text or remove these references and replace them with relevant current references. Any changes to the reference list should be mentioned in the rebuttal letter that accompanies your revised manuscript. If you need to cite a retracted article, indicate the article’s retracted status in the References list and also include a citation and full reference for the retraction notice.

**Response:** Thank you so much. We have ensured that all references are correct and complete, and we didn’t cite any retracted articles in our manuscript.

Reviewer #2 comments: The manuscript is much improved following the revisions. Two final minor points to consider:

**Response:** Thank you for your positive feedback and for acknowledging the improvements in the manuscript. We appreciate your careful review and will address the comments below.

Is there an age or timing cut-off for the early life risk factors? Can these be measured at the same time as the outcomes?

**Response:** In our study, we define early childhood as up to 8 years of age, following the WHO guidelines. We have included cohort, case-control, and cross-sectional studies in our systematic review. Therefore, early childhood risk factors are considered as risk factors reported for children less than 8 years, which corresponds to the period of outcome assessment. In cross-sectional studies, it is possible that risk factors and outcomes are measured at the same time. In cohort and case-control studies, however, risk factors are typically measured at baseline and/or during follow-up, allowing for a temporal separation between risk factors and outcomes. Therefore, we will be acknowledging this inherent limitation in our study.

I am still struggling to understand how the authors will delineate individual risk factors such as ‘personal behaviours’ p. 4 line 104 from the social-emotional development outcomes. These will be the same measures in many cases. Unless this is no longer one of the risk factors being examined, but rather just part of the theoretical model? This may become an issue at the time of data extraction.

**Response:** Thank you for your insightful feedback. We acknowledge the importance of clearly delineating ‘personal behaviours’ from ‘social-emotional’ developmental outcomes. We have revised the personal behaviour-related factors into lifestyle and behavioural factors, which include the presence of childhood books and storytelling, toys, childhood punishment, mineral and vitamin supplementation, excessive screen time, physical activity, sleeping patterns, etc. While social-emotional development is one of the domains of developmental outcome reported in most developmental measurement tools as we have observed in the preliminary literature review on the topic.

Response to Reviewer #4 comments: Overall, the protocol is well written. I have a few minor suggestions that I believe could improve the manuscript:

**Response:** Thank you for the positive feedback on our protocol. We appreciate your suggestions for improvement.

1. In the comparison group, the definition of "low level of exposure to the risk factors" should be clarified.

**Response:** In our study, "the comparators are children with no or low level of exposure to the risk factors" refers to individuals or groups who have minimal exposure to the identified risk factors compared to the general population; this will be determined based on specific thresholds or criteria established in the included studies. Some studies might have exposure levels like low, medium and high; in that case, we will use low levels of exposure as a comparator, particularly in environmental exposures.

2. In the JBI quality assessment, it would be better if the author predefined what constitutes a high score for considering a specific study as high quality. Additionally, the author should specify whether extremely low-quality studies will be excluded, and if so, what cutoff point is used to exclude them. Some literature suggests using >75% as high quality and <50% as low quality to exclude studies.

**Response:** There are still arguments to include or exclude the studies with low-quality scores. Hence, we plan to do a sensitivity analysis if the data will allow us, or we will conduct a descriptive analysis based on the study JBI quality score rather than excluding studies with low quality. We have delineated the score into high, moderate and low for >75, between 50-75 and <50, respectively.

3. There is inconsistency throughout the manuscript regarding whether this review includes a meta-analysis. In some sections, the author refers to both a systematic review and a meta-analysis. If a meta-analysis is included, the methods of synthesis need to be better planned.

**Response:** Previously, we thought to conduct a meta-analysis, but now after we had a preliminary literature review, we thought the data would not allow us to conduct a meta-analysis; rather, informed by the socioecological model, we will undertake narrative synthesis to comprehensively summarise the risk factors. We have revised the manuscript, and we have now removed the term meta-analysis.

4. In the Data Synthesis section, lines 213-218, the statement "The Socioecological model provides..." is irrelevant to this section, as it has already been described in the Introduction.

**Response:** We have now removed the redundant statement about the Socioecological model from the Data Synthesis section.

5. If possible, I suggest the author consider approaches to evaluate the certainty of the evidence that will be generated from this systematic review (e.g., the GRADE approach).

**Response:** Thank you for your valuable suggestion. We agree that evaluating the certainty of the evidence generated from our systematic review is crucial for providing robust and transparent conclusions. Hence, we plan to use the GRADE (Grading of Recommendations, Assessment, Development, and Evaluations) approach in our analysis to assess the quality of evidence across the included studies.

6. In the Discussion section, it would be better if the author discussed the anticipated limitations of this study protocol (e.g., any limitations of the Socioecological model) rather than repeating what was stated in the Introduction.

**Response:** We have revised to include the anticipated limitations of our systematic review in the discussion sections as follows: “One of the anticipated limitations of our systematic review is the heterogeneity in early childhood developmental assessment tools, which could lead to variability in how developmental outcomes and their domains are reported. This diversity might introduce challenges in synthesising the data and drawing consistent conclusions across studies. To address this, we will employ the socioecological model, which provides a systematic group of risk factors, enhancing a multilevel targeting approach.
